# Use of Pasireotide in Acromegaly: Clinical Experiences From a Series of Patients in Qatar

**DOI:** 10.1155/crie/6400298

**Published:** 2025-11-16

**Authors:** Tarik Elhadd, Zeinab Dabbous, Elabbass A. Abdelmahmuod, Zaina Rohani, Yaman Al Kailani

**Affiliations:** ^1^Endocrine Section, Department of Medicine, Hamad Medical Corporation, Doha, Qatar; ^2^Department of Neuro-Radiology, Hamad Medical Corporation, Doha, Qatar

**Keywords:** acromegaly, case series, pasireotide, patient journey, Qatar

## Abstract

**Background:**

Acromegaly, a rare endocrine disorder characterised by excess growth hormone (GH) and insulin-like growth factor 1 (IGF-1), is often due to GH-secreting pituitary adenomas. Pasireotide, a second-generation somatostatin receptor ligand (SRL), binds to multiple somatostatin receptors (SSTRs) and offers a promising alternative for patients unresponsive to first-generation SRLs like octreotide and lanreotide.

**Case Series:**

This report examines five acromegaly patients treated with pasireotide in Qatar after the failure of a first-generation SRL to normalise IFG-1 levels. Patient 1, a 39-year-old male with hyperprolactinaemi+a and acromegaly who underwent multiple therapies including surgery and radiotherapy, showed tumour size reduction and IGF-1 control with pasireotide. Patient 2, 48-year-old male with a significant macroadenoma and prior cabergoline treatment, achieved partial biochemical control. He developed Type 2 diabetes mellitus, which was managed with metformin/sitagliptin. Patient 3, a 41-year-old male, experienced dramatic symptom resolution and weight loss after switching to pasireotide, significantly improving his quality of life, with a reduction in tumour size. Patient 4, 52-year-old female, despite initial side effects on pasireotide, achieved normalisation in IGF-1 levels and resolution of active symptoms, with a significant reduction in tumour size. Patient 5, a 38-year-old female, after persistent elevation of IGF-1 on octreotide, responded well to pasireotide with a significant reduction in IGF-1. In all five cases switching to pasireotide demonstrated marked efficacy by normalising IGF-1 and eliminating acromegaly symptoms within the first months of treatment. Four out of these five patients showed reduction in tumour size.

**Conclusion:**

This case series corroborates the findings from previous studies, adding insight into treatment challenges and benefits experienced by this heterogeneous group of patients on pasireotide.

**Summary**

• Pasireotide demonstrated efficacy in normalising insulin-like growth factor 1 (IGF-1) levels and controlling acromegaly symptoms in patients who were unresponsive to first-generation somatostatin receptor ligands (SRLs).
• Four out of five patients showed significant tumour size reduction with pasireotide treatment, highlighting a potent anti-tumour activity.
• Pasireotide was generally well-tolerated, with manageable side effects, including expected hyperglycaemia in some patients.

## 1. Background

Acromegaly is a rare endocrine disorder, with a prevalence ranging from 2.8 to 13.7 cases per 100,000 people [1]. It is typically characterised by elevated levels of growth hormone (GH) and insulin-like growth factor 1 (IGF-1), usually due to a GH-secreting adenoma [2]. Transsphenoidal pituitary surgery (TSS) is the first-line therapy for acromegaly, as it can effectively alleviate the mass effects of the tumour. However, for some other patients, where surgery may not be deemed appropriate or if they do not wish for surgery other options like somatostatin receptor ligands (SRLs) may be the best option. For patients with recurrent or persistent disease, therapeutic options including repeat surgery, radiotherapy, and medical therapy [3]. Medical treatments include SRLs, dopamine agonists alone, or in combination with SRLs, and GH receptor antagonists [3]. First-generation SRLs, such as octreotide and lanreotide, bind primarily to somatostatin receptor-2 (SSTR2). However, their effectiveness is suboptimal, with IGF1 normalisation reported in about 30%–50% of patients [4–6]. Pasireotide, a second-generation SRL, binds to multiple SSTRs and has shown promise for patients who do not achieve disease control with first-generation SRLs [7–10]. Although the effects of treatment with pasireotide long-acting release (LAR) have been well described in clinical studies, real-world practice evidence of the effectiveness and safety of this agent is still limited, especially in the long term. This case series presents reports from five patients in Qatar treated with pasireotide for active acromegaly, detailing their responses to therapy. These reports may enhance understanding of diverse or understudied acromegaly populations and help local clinicians who may be unfamiliar with this drug to optimise its use, and following from that, help to improve the care of this patient population.

## 2. Case Series

### 2.1. Patient 1

**Figure 1:**
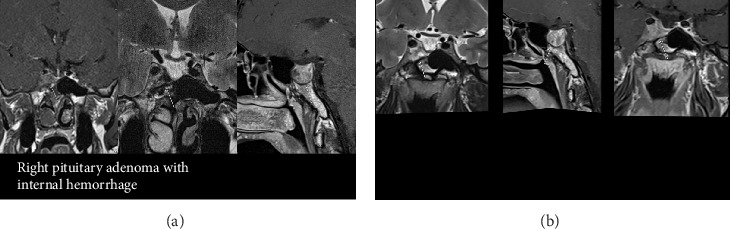
(A) Dcember 2021 MRI showing a pituitary tumour dimensions of 23.8 mm × 18.9 mm ×18 mm. (B) MRI December 2023 showing greater brightness of the pituitary tumour and reduction in size 21.3 mm × 18.3 mm × 17 mm.

A 39-year-old Jordanian male, diagnosed with acromegaly in 2013 following symptoms of severe headache, general weakness and coarseness of features. An initial magnetic resonance imaging (MRI) revealed a 15 mm × 17 mm × 24 mm macroadenoma. He underwent transsphenoidal hypophysectomy in 2013. Immune-histochemical analysis confirmed a GH-secreting somatotrophinoma. Two years postsurgery in 2015, medical treatment with the SRL lanreotide 120 mg/month was initiated due to active acromegaly. In 2017, he received radiotherapy (54 Gy/30 sessions). An MRI in 2020 revealed a residual tumour, unchanged right sellar and remnants in the right cavernous sinus. There was no hypopituitarism as a sequelae to his surgery and radiotherapy. In 2021, he moved to Qatar and treatment was switched from lanreotide 120 mg/month to octreotide (Sandostatin LAR), as lanreotide was not available in Qatar. However, his IGF-1 remained elevated despite continuous SRL therapy (Table 1). His December 2021 MRI showed residual adenoma in right cavernous sinus with tumour dimensions of 23.8 mm × 18.9 mm × 18 mm (Figure 1A). He was proposed surgery but refused. Despite an increase in octreotide dose to 40 mg/month, IGF-1 remained elevated, so he was switched to pasireotide 40 mg in June 2023. On MRI, tumour dimensions in December 2023 were 21.3 mm × 18.3 mm × 17 mm, signal intensity on *T*_2_ images showed greater brightness than previously and illustrated a reduction in the tumour size (Figure 1B). His HbA1c was 5.9%. His IGF-1 level normalised following pasireotide therapy (Table 1). On follow up till May 2025 his IGF-1 remained within normal reference range (Table 1), and MRI pituitary showed stable size of the remnant.

In this 40-year-old patient, for whom a second surgery was declined, treatment with pasireotide LAR has shown promising results, leading to a reduction in tumour size as evidenced by the latest MRI findings, without significantly impacting his glucometabolic status.

### 2.2. Patient 2

A 48-year-old Egyptian male presented in Egypt in 2017 for obesity and was found to have a 35 mm × 35 mm pituitary macroadenoma. He was treated with cabergoline for 6 months on the assumption that it was a prolactinoma. He presented in our clinic in 2019 with no remarkable features on general examination but was revealed to have hyperprolactinaemia, acromegaly, secondary hypothyroidism and secondary hypogonadism. The intrasellar mass was 29 mm in maximum axial diameters, encroaching on the left side of the optic chiasm. Neurosurgery was not possible because of the extension of the macroadenoma into the cavernous sinus. As octreotide was not affordable for this patient, he was started on cabergoline and the dose increased to 0.5 mg daily, which resulted in normalisation of prolactin levels, but IGF-1 levels remained high (Table 1). Octreotide as long-acting preparation, ‘Sandostatin-LAR', provided by a charitable institution, was initiated at a dose of 20 mg/monthly initially, later titrated to 30 mg and then 40 mg per month. This failed in normalising IGF-1, which in September 2022 was high, at 525 µg/L (Table 1). Repeated MRIs every 6 months showed no significant interval changes in the size, morphology or signal characteristics of the sellar/suprasellar macroadenoma. The patient received two sessions of radiotherapy, but declined further sessions for fear of side effects. This may provide explanation for his persistent and active disease.

In October 2022, he was switched to pasireotide 40 mg/month. After 3 months, his prolactin, GH and IGF-1 levels improved. Serial IGF-1 levels confirmed sustained normalisation (Table 1) and latest IGF-1 was 178 µg/L (normal range for age: 79–214 µg/L). Emergence of Type 2 diabetes mellitus was controlled with metformin/sitagliptin. MRI, in December 2023, there was no change in tumour size. On regular follow up to June 2025 his IGF-1 remained stable (Table 1), and MRI scan showed no further increase in size.

In this 48-year-old patient, for whom neurosurgery was not feasible due to the macroadenoma's extension, treatment with pasireotide LAR successfully improved prolactin, GH and normalised IGF-1 levels, while tumour size remained stable. His Type 2 diabetes is well-managed with metformin/sitagliptin.

### 2.3. Patient 3

**Figure 2:**
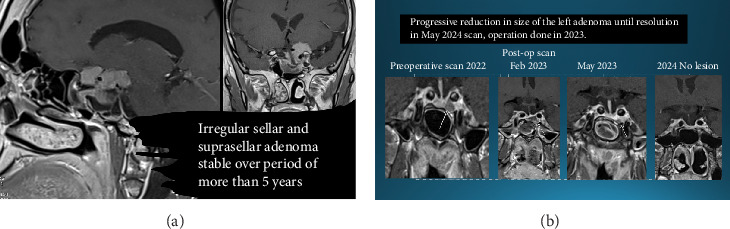
(A) MRI May 2023. (B) MRI May 2024.

A 41-year-old Yemeni man was diagnosed with acromegaly in 2016 following a tonsillectomy. In 2018, he underwent TSS, which was repeated in February 2023, for significant increase in the residual tumour size and active disease. IGF-1 remained high, with symptoms of active disease, including excessive sweating, arthritis and tiredness. Residual pituitary function was suggestive of partial hypopituitarism in addition to active clinical and biochemical acromegaly. He started on octreotide, but it was discontinued for financial reasons. IGF-1 was persistently elevated following two surgeries and treatment with first-generation SRLs (Table 1). An MRI scan in May 2023 following second surgery in February 2023 showed a residual tumour (Figure 2A). In August 2023, he was started on pasireotide 40 mg. By December 2023, his acromegalic features and symptoms resolved, he lost 14 kg and his quality of life improved significantly, IGF-1 improved significantly. The dose of pasireotide was increased to 60 mg in January 2024. IGF-1 measurement in May 2024 was even lower, at 87 µg/L. MRI in May 2024 showed complete resolution of the residual tumour (Figure 2B). Follow up to May 2025 showed stable disease (Table 1). AS per remarkable outcome, patient was keen to continue on the same dose of pasireotide.

In this 41-year-old patient, in whom two surgeries did not achieve disease control, treatment with pasireotide LAR led to the resolution of acromegalic features and symptoms, significantly improving his quality of life, with a substantial weight loss of 14 kg (weight was 95 kg in July 2023 prior to starting treatment with pasireotide and dropped gradually to 81 kg by the end of 2024). The reason behind that we hypothesise was in relation to complete change of lifestyle and resolution of symptoms of active acromegaly. The patient has complete resolution of his acromegaly, IGF-1 plummeted. His debilitating symptoms of arthralgia and arthritis resolved, and he was able to exercise. In addition, complete regression of the tumour remnant was achieved.

### 2.4. Patient 4

**Figure 3:**
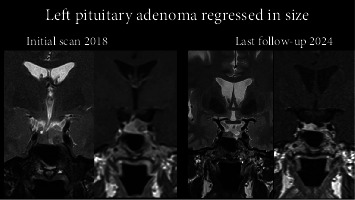
MRI before (left), and after (right) pasireotide therapy showing reduction in size of the pituitary adenoma.

A 52-year-old Qatari woman was diagnosed in 2017 with acromegaly after an initial referral to our clinic for high prolactin, complaining of breast engorgement and irregular periods. She was noted to have subtle features of acromegaly including minor changes in facial features and early prognathism. The patient had no symptoms of acromegaly whatsoever. The diagnosis was confirmed considering the subtle features of acromegaly combined with the high IGF-1 when her pituitary function was assessed. In 2018, MRI revealed a 9 mm × 8 mm microadenoma (Figure 3) and her IGF-1 level was elevated. The patient was offered surgery, however, when this was attempted, she could not be intubated, and the procedure was abandoned. Subsequently, the patient declined the surgical option as she was scared following the first attempt. Treatment was started with octreotide as Sandostatin LAR 20 mg per month, and dose was later increased to 30 mg per month. Cabergoline 0.5 mg weekly was added, but this failed to normalise IGF-1. The microadenoma appeared stable over time. In July 2023 she was initiated on pasireotide 40 mg/month. Her IGF-1 levels were normalised, with resolution of all signs of active acromegaly. She experienced initial side effects following the first injection, but no long-term issues were noted. No negative effect on her glycaemic status was exhibited in the wake of pasireotide therapy. An MRI scan prior to pasireotide therapy had shown failure of reduction in the size of the tumour when she was on combined cabergoline and first-generation SRL therapy. However, 6 months into pasireotide treatment, the tumour size had decreased in size from 9 mm × 7 mm × 7 mm (craniocaudal, transverse, anteroposterior measurements) to 7 mm × 3 mm × 6 mm in the above dimensions respectively (Figure 3). On regular follow up to July 2025, IGF-1 remained within normal (Table 1) and MRI showed stable remnant.

In this 52-year-old patient, in whom combined treatment with octreotide and cabergoline failed to normalise IGF-1 levels, switching to pasireotide LAR successfully normalised her IGF-1 levels and resolved all signs of active acromegaly, with only transient side effects following the first injection, with an added benefit of tumour size reduction not achieved before by the combination of a first-generation SRL and dopamine agonist therapy.

### 2.5. Patient 5

**Figure 4:**
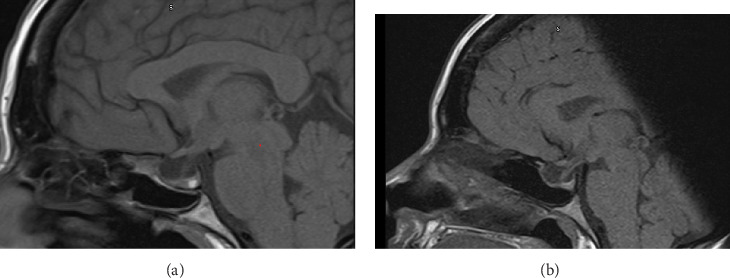
(A) Pituitary tumour encasing the L cavernous sinus before pasireotide therapy and (B) reduction of the tumour size following pasireotide therapy.

A 40-year-old Indian woman was diagnosed with acromegaly in a private clinic in 2021 with a history of headache and a gradual coarsening of her facial appearance with clearly recognisable acromegalic facies. She has a background of Type 2 diabetes and hypertension. She had a very high level of IGF-1 and a macroadenoma of 27 mm in maximum diameter encasing of the internal carotid and some degree of suprasellar extension abutting of optic chiasm. Following a TSS in 2022, residual tumour persisted (Figure 4A) and with persistent active acromegaly symptoms. Despite treatment with octreotide 20 mg/month, increased to 30 mg, active symptoms persisted and her IGF-1 remained elevated (Table 1). In November 2023, she was switched to pasireotide 40 mg. At her January 2024 follow-up, her IGF-1 had normalised. Minor and transient side effects during the first 2 days of pasireotide were experienced, but have not been noticed since. She reported losing weight and that all her active symptoms of acromegaly have been resolved. Repeat MRI scan showed significant reduction in the size of the residual tumour encasing the Lt Cavernous Sinus (Figure 4B). There was also some minor deterioration in her glycaemic control, which was managed effectively by adjusting her medication. Up to recent follow up in 2025, the IGF-1 remained within the normal reference range (Table 1) and repeat MRI scan showed stable remnant.

In this 40-year-old patient, in whom surgery left a residual tumour and octreotide failed to control her disease, switching to pasireotide LAR successfully normalised her IGF-1 levels and resolved all active symptoms of acromegaly, with an added benefit of reduction in the size of the residual pituitary tumour, with only minor and transient side effects during the first 2 days of treatment.

## 3. Discussion

This case series includes five very heterogeneous patients with active symptoms of acromegaly and pituitary adenomas. Irrespective of previous TSS or radiotherapy status, in all cases patients had failed a first-generation SRL. Multiple surgeries and radiotherapy may fail to control active acromegaly when there is residual disease, which is a well-recognised feature in these patients with refractory disease. Most patients, if not all, were reporting persistent symptoms and no significant tumour or tumour remnant shrinkage and were eventually all switched to the second-generation SRL, pasireotide, which is available in Qatar for this indication. All five patients reported marked improvements or complete resolution of their active acromegaly symptoms within months of switching to pasireotide. This finding is consistent with previous clinical studies comparing pasireotide and octreotide. A study in 358 medically-naïve patients with acromegaly, pasireotide vs. octreotide achieved normal GH levels (31% vs. 19%), and normal IGF-1 levels (35% vs. 20%) [8]. Studies have shown that pasireotide can achieve better normalisation of GH and IGF-1 levels than comparative SRLs, as well as significant tumour volume reduction in many patients. In a head-to-head comparative study, pasireotide 40 and 60 mg given for 24 weeks were superior to both octreotide 30 mg and lanreotide 120 mg in achieving normalisation of IGF-1 and reduction of GH to under 2.5 μg/L [7]. In a cross-over study of acromegaly patients inadequately controlled on octreotide 30 mg, pasireotide was more effective in controlling parameters of acromegaly activity (GH < 2.5 μg/L, normal IGF-1) [10]. In addition, in Phase III trials, pasireotide has demonstrated sustained inhibition of GH and IGF-1 in patients with active acromegaly for up to 25 months [11].

Pasireotide's potent anti-tumour activity in acromegaly has been documented in both clinical and preclinical studies. In a cohort of 358 medical treatment-naïve patients with acromegaly, tumour volume reduction was achieved in 81% of patients with acromegaly compared with 77% in those receiving octreotide LAR [9]. In rat GH secreting tumour cell lines, in studies using octreotide and pasireotide looking at cell viability, cell cycle, apoptosis, GH secretion and tumour induced angiogenesis, pasireotide showed more potent anti-tumour activity than octreotide [12]. It is noteworthy that, four out of our five patients all had significant reduction in their tumour size. Furthermore, one patient (Patient 2), had his residual tumour completely vanished after 1-year of treatment and showed dramatic improvement in weight and acromegalic features. The tumour reduction effect of pasireotide has been suggested by Daly et al. [13] to be related to its effect on the germline mutation of Aryl hydrocarbon receptor interacting protein (AIP). Mutations in the AIP has been shown to be behind the aggressiveness of certain pituitary tumours causing acromegaly especially in younger or in familial cases, with subsequent poor response to first generation SRLs [14, 15]. Our case series lends support for the potent anti-tumour activity of pasireotide.

The finding that the overwhelming majority of patients lost significant amounts of weight on pasireotide is interesting as, although this effect is established and pronounced in patients with Cushing's disease [16], it has not been widely reported in acromegaly and may represent a significant patient benefit.

Pasireotide is known to be associated with a higher risk of hyperglycaemia in patients categorised as diabetic or prediabetic before treatment [17]. In the present case series, two patients developed diabetes, both of which were successfully managed with antidiabetics. The third one with pre-existing diabetes had initially worsening glycaemic control, but it was effectively managed with medication. A good safety profile and long-term tolerability were reported for all patients. This is consistent with previous evidence on long-term treatment with pasireotide-LAR [18] and with data showing that pasireotide-LAR monotherapy is more cost-effective compared with combination treatment and has an advantage when there are concerns of tumour growth [19].

## 4. Conclusions

This case series highlights the therapeutic potential of pasireotide in managing acromegaly in patients unresponsive to first-generation SRLs. Pasireotide demonstrated efficacy in reducing IGF-1 levels and controlling disease activity and markedly improved these patients' quality of life, in four out of five cases helping them lose weight and eliminating the symptoms of active disease. Furthermore, in four out of five patients, the tumour size reductions were clinically meaningful benefit. These findings contribute to the growing body of evidence supporting pasireotide as a valuable treatment option for treatment-resistant acromegaly.

## Figures and Tables

**Table 1:** Levels of IGF-1 in the five patients.

Patient 1	October 22	April 23^a^	June 23	September 23	May 24	September 24	February 25	June 25							
IGF-1 (µg/L)^b^	386	403	182	108	223	202	221	234							
Patient 2	September 22	January 23	April 23^a^	October 23	January 24	February 24	August 24	November 24	February 25	August 25					
IGF-1 (µg/L)	525	233	236	176	178	212	134	204	207	134					
Patient 3	November 16	May 17	February 18	March 19	January 20	June 21	December 21	August 22	February 23	April 23^a^	November 23	January 24	May 24	November 24	May 25
IGF-1 (µg/L)	413	444	520	727	632	714	818	727	419	465	118	176	87	147	208
Patient 4	April 18	June 19	November 19	August 20	June 21	March 22	March 23a	August 23	December 23	January 24	July 24	November 24	May 25		
IGF-1 (µg/L)	346	417	452	420	382	382	313	158	147	118	159	228	119		
Patient 5	February 22	September 22	December 22	May 23a	January 24	March 24	September 24	June 25	September 25						
IGF-1 (µg/L)	822	619	362	353	134	144	93	192	124						

^a^Indicates the start of pasireotide prior to that date.

^b^The normal reference range for IGF-1 varies according to age and sex, but generally it is 92–224 µg/L for most patients.

## Data Availability

All relevant clinical data is provided in the publication.
